# Supplementary material for: 0D van der Waals interfacial ferroelectricity
Source: Nat Commun. 2023 Oct 31;14:5578. doi: 10.1038/s41467-023-41045-8 (PMC10618478; doi:10.1038/s41467-023-41045-8)
Supplement: Supplementary file 1 — Supplementary Information [file 41467_2023_41045_MOESM1_ESM.pdf]

Supplementary information for

0D van der Waals interfacial ferroelectricity

Yue Niu, Lei Li, Zhiying Qi, Hein Htet Aung, Xinyi Han, Reshef Tenne, Yugui Yao, Alla Zak, Yao Guo

Email: yaoguo@bit.edu.cn

Table of content

Supplementary Fig. 1. Schematic diagram of different dimensions of the interface via combinations of vdW materials 3

Supplementary Fig. 2. WS_2 nanotubes synthesized by the two-step reaction route 4

Supplementary Fig. 3. Structural characterization of WS_2 nanotube 5

Supplementary Fig. 4. Schematic of device fabrication 6

Supplementary Fig. 5. Maximum pressure intensity of the stacked WS_2 nanotubes as a function of the height difference 7

Supplementary Fig. 6. The vdW interfacial ferroelectricity domain size of different materials 8

Supplementary Fig. 7. Equivalent circuit of the device 9

Supplementary Fig. 8. Fitting of the Arrhenius plot, ln($I/T^{3/2}$) vs 1000/T 10

Supplementary Fig. 9. Memory function of WS_2 nanotube device 11

Supplementary Fig. 10. Theoretical calculation of built-in interlayer potential 12

Supplementary Fig. 11. Number of atoms of the core part of different devices 13

Supplementary Fig. 12. The temperature distribution of the simulated device 14

Supplementary Fig. 13. The programmed and measured pulse waveform 15

Supplementary Fig. 14. Retention characteristics of the ON (black) and OFF (red) states 16

Supplementary Fig. 15. Endurance of resistive switching with the pulses 17

Supplementary Table 1. Endurance of ferroelectric devices 18

Supplementary Fig. 16. Absorption spectra of the pristine WS_2 nanotubes and $BiFeO_3$ 19

Supplementary Fig. 17. Laser power dependence of the photocurrent density 20

Supplementary Fig. 18. The simulated optical electric field distribution of WS_2 nanotubes crossbar 21

Supplementary Fig. 19. Maximum electric displacement as a function of irradiation wavelength 22

References 23

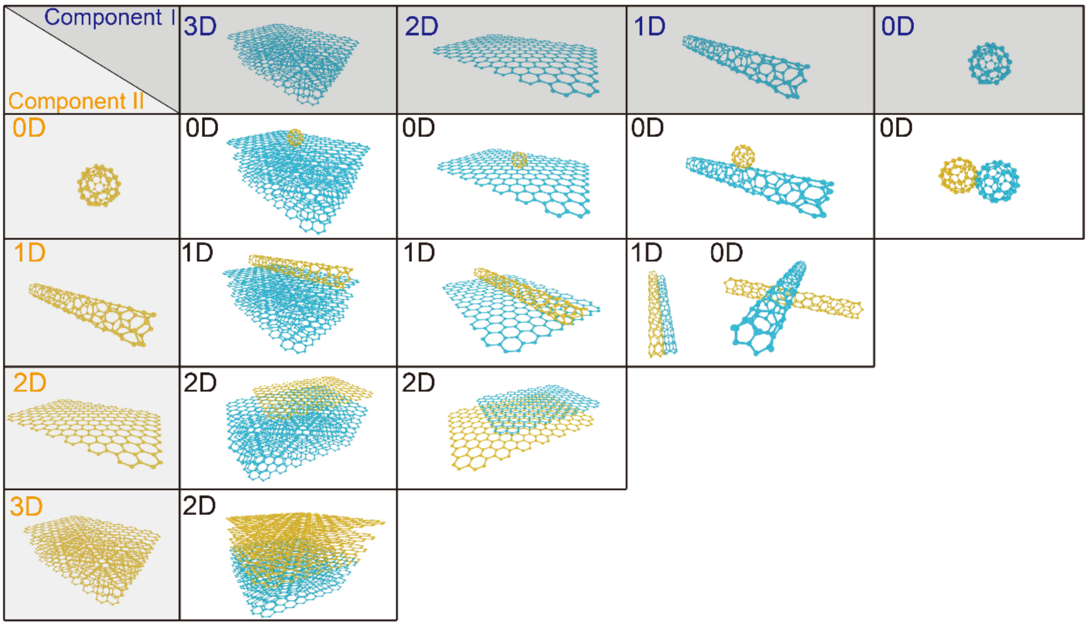

Supplementary Fig. 1 | Schematic diagram of different dimensions of the interface via combinations of vdW materials.

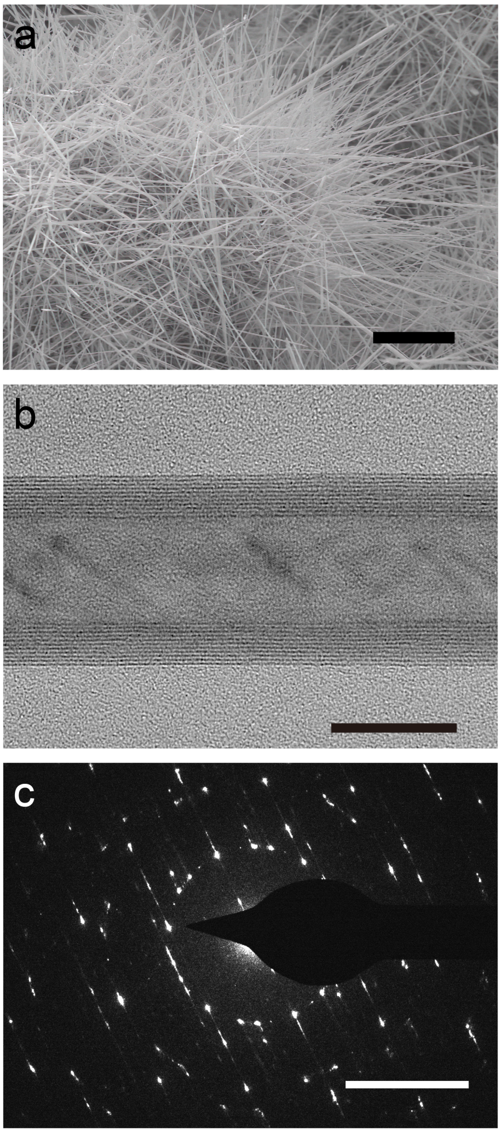

Supplementary Fig. 2 | WS_2 nanotubes synthesized by the two-step reaction route. **a,** Scanning electron microscopy image of the WS_2 nanotubes. The scale bar is 5 μm. **b,** Transmission electron microscopy image of a multiwall WS_2 nanotube. The scale bar is 20 nm. **c,** Electron diffraction pattern of the WS_2 nanotube. The scale bar is 5 1/nm.

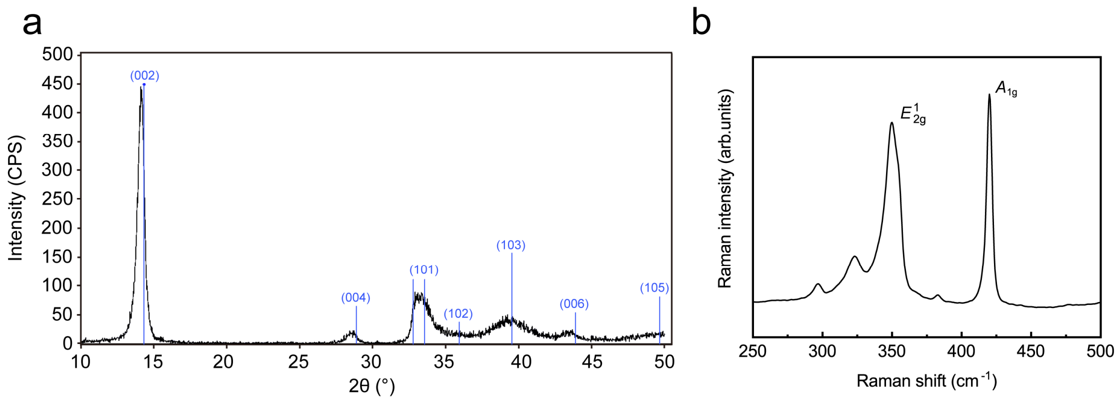

Supplementary Fig. 3 | Structural characterization of WS_2 nanotube. a, The x-ray diffraction of the WS_2 nanotubes. The peak of the (002) plane of the nanotubes shows an interlayer distance of 6.4 Å. **b,** The Raman spectrum of the WS_2 nanotube. The $E^1{}_{2g}$ mode involves an in-phase vibration of the W atoms with respect to the S atoms vibrating in the opposite direction in-phase, while the A_{1g} mode arises from the S atoms moving in in-phase and in out-of-plane directions.

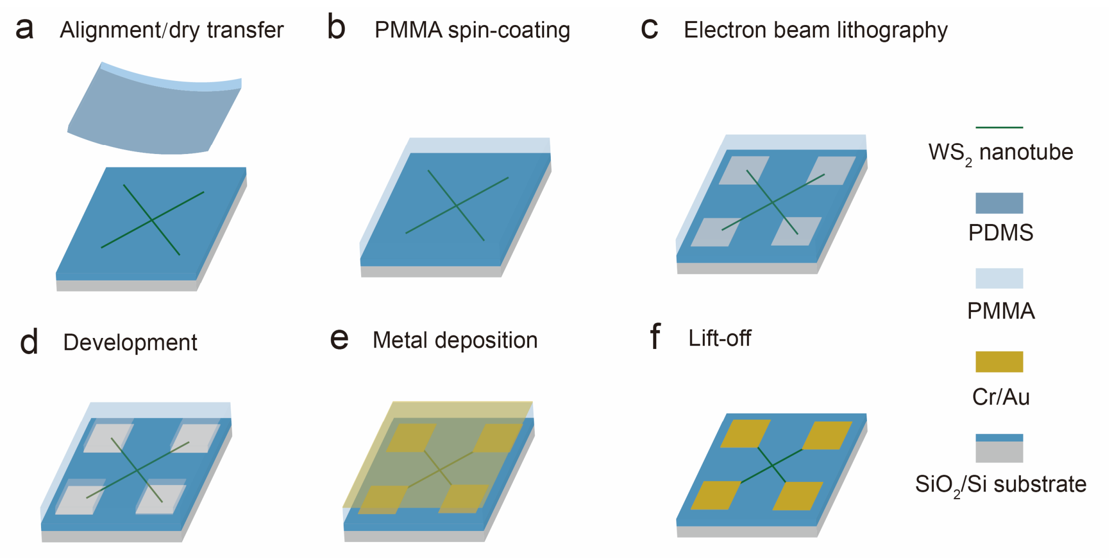

Supplementary Fig. 4 | Schematic of device fabrication. a, The WS_2 nanotubes were transferred to Si oxide substrate. **b,** A layer of PMMA was coated on the surface. **c** Fabrication of the electrodes by electron beam lithography. **d,** The development process of the device. **e,** The contacting electrodes were deposited by the e-beam evaporation. **f,** The redundant metal was removed by the lift-off process in acetone.

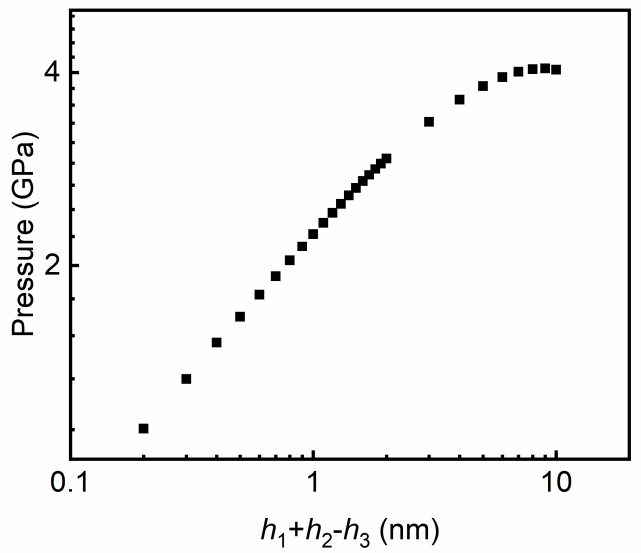

Supplementary Fig. 5 | Maximum pressure intensity of the stacked WS_2 nanotubes as a function of the height difference.

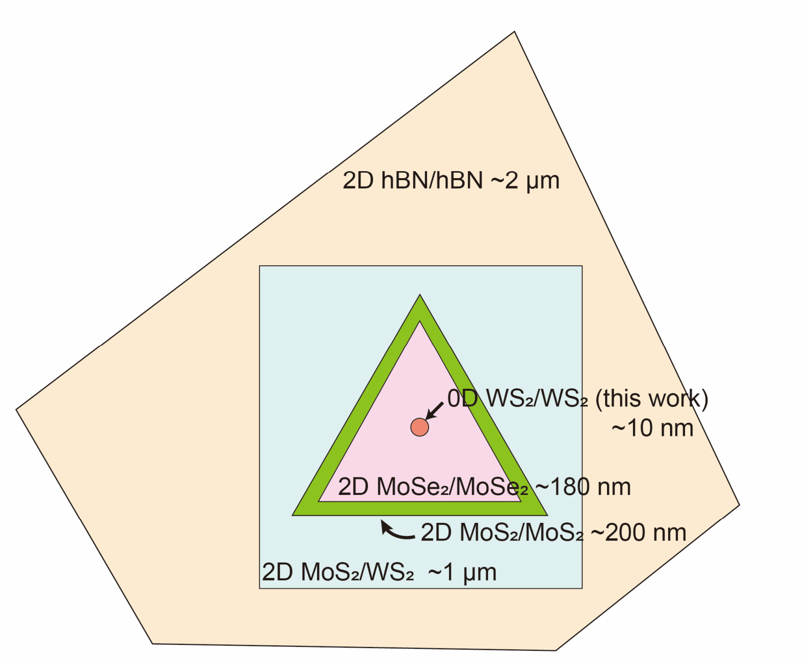

Supplementary Fig. 6 | The vdW interfacial ferroelectricity domain size of different materials. The experimental values of 2D $MoSe_2$/$MoSe_2$ interface[1], 2D MoS_2/MoS_2 interface[2], 2D MoS_2/WS_2 interface[3] and 2D hBN/hBN interface[4] were extracted from references. Non-proportional scale for visualization.

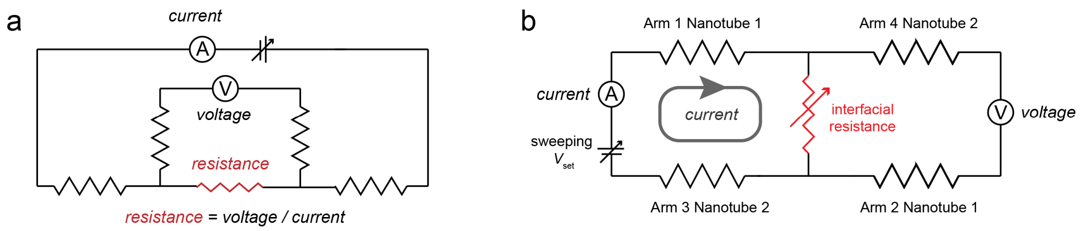

Supplementary Fig. 7 | Equivalent circuit of the device. a, Schematic diagram of the four-terminal measurement. **b,** Schematic circuit diagram of the four-terminal measurement applied in this work.

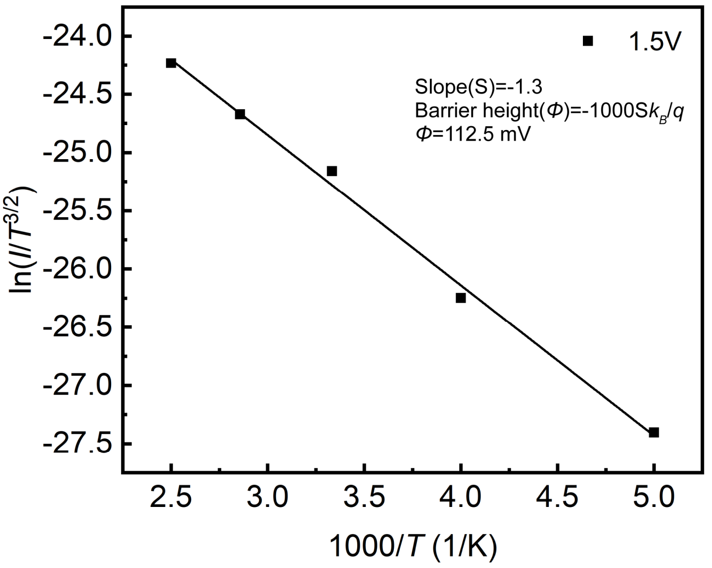

Supplementary Fig. 8 | Fitting of the Arrhenius plot, ln($I/T^{3/2}$) vs 1000/T.

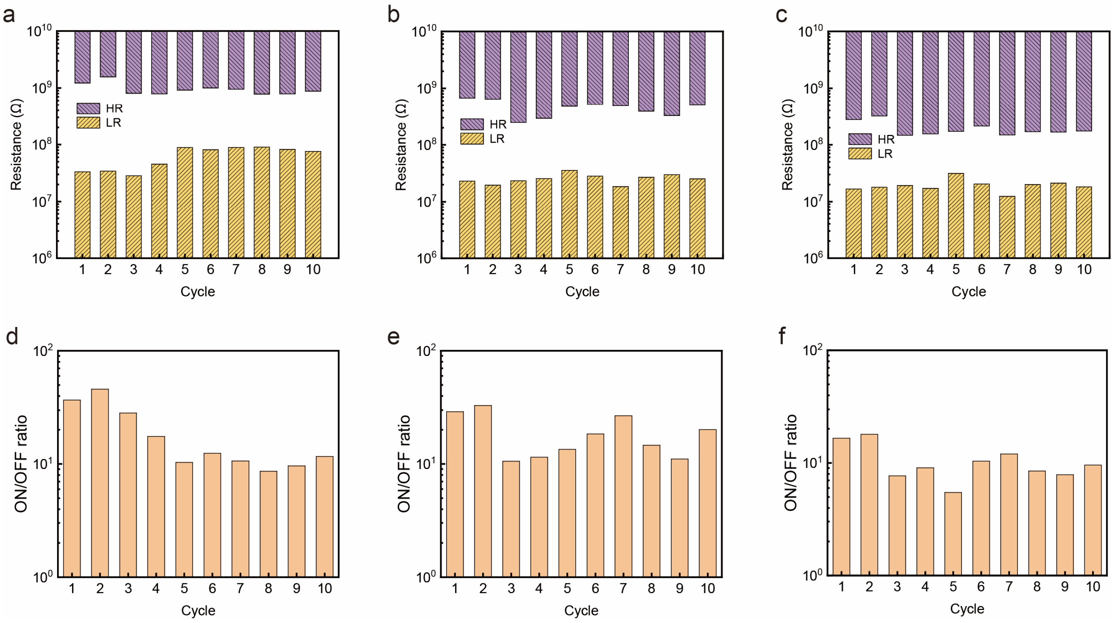

Supplementary Fig. 9 | Memory function of WS_2 nanotube device. a-c, The measured ON-state (yellow bars) and OFF-state (purple bars) resistances of the device, read at V=0.5 V(a), V=1.5 V(b) and V=2 V(c). The ON/OFF state corresponding to low /high resistance (LR/HR) state respectively. **d-f,** The ON/OFF ratio of the device, read at V=0.5 V(d), V=1.5 V(e) and V=2 V(f).

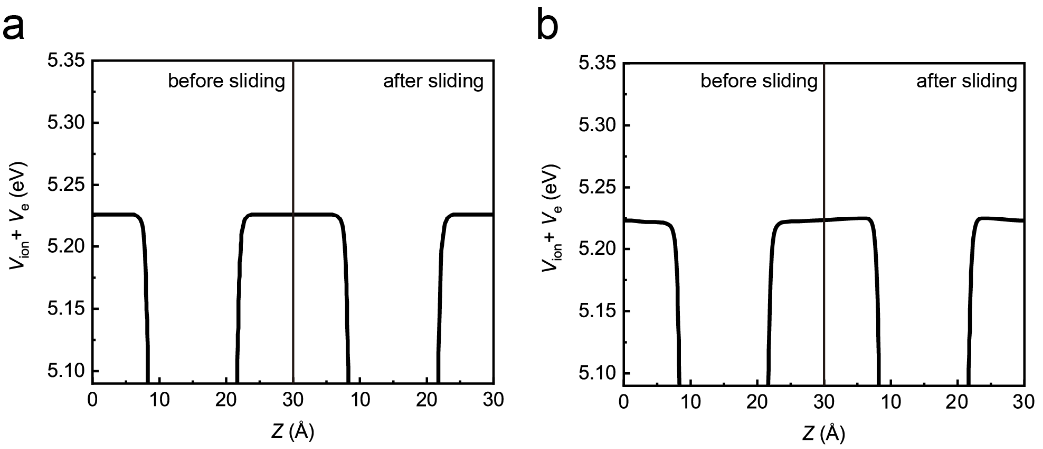

Supplementary Fig. 10 | Theoretical calculation of built-in interlayer potential. Potential landscape ($V_{ion} + V_e$) of bilayer WS_2 in **a,** parallel-stacked configuration and **b,** antiparallel-stacked configuration. The results of interlayer potential were extracted from ref.[1].

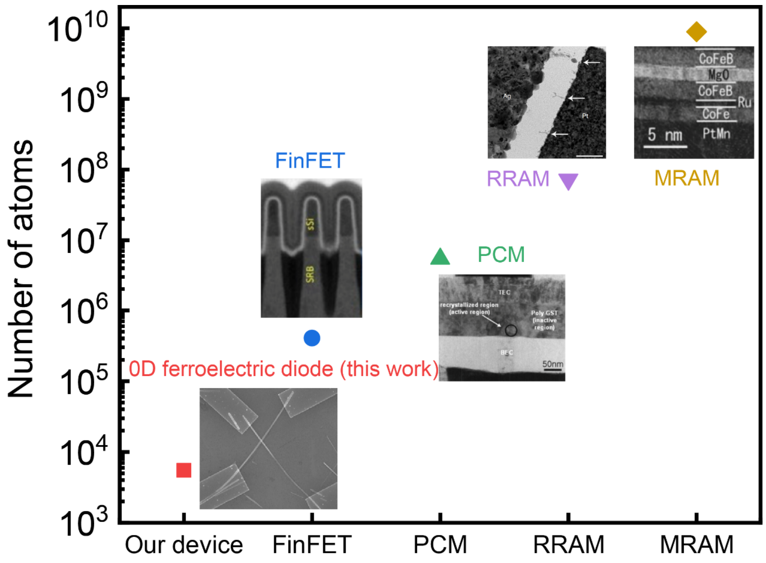

Supplementary Fig. 11 | Number of atoms of the core part of different devices. The numbers of FinFET[5], PCM[6], RRAM[7] and MRAM[8] were extracted from the literature.

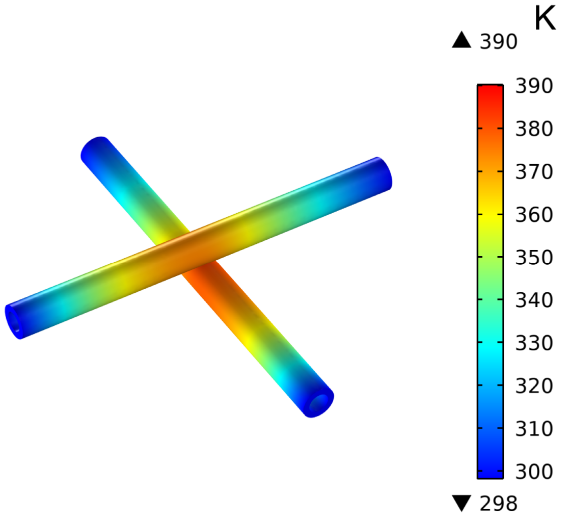

Supplementary Fig. 12 | The temperature distribution of the simulated device.

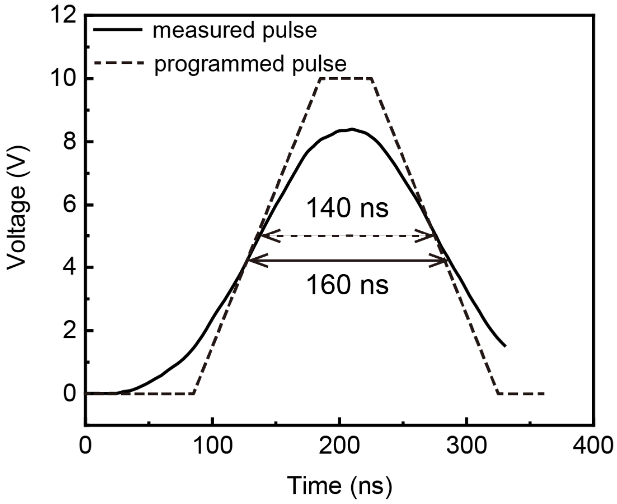

Supplementary Fig. 13 | The programmed and measured pulse waveform.

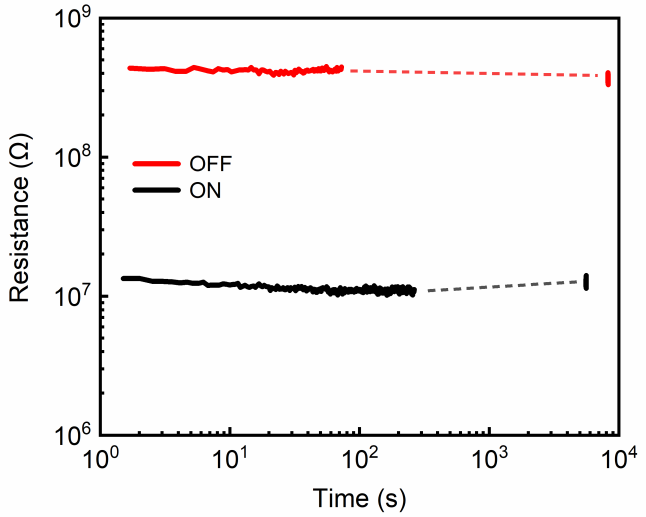

Supplementary Fig. 14 | Retention characteristics of the ON (black) and OFF (red) states.

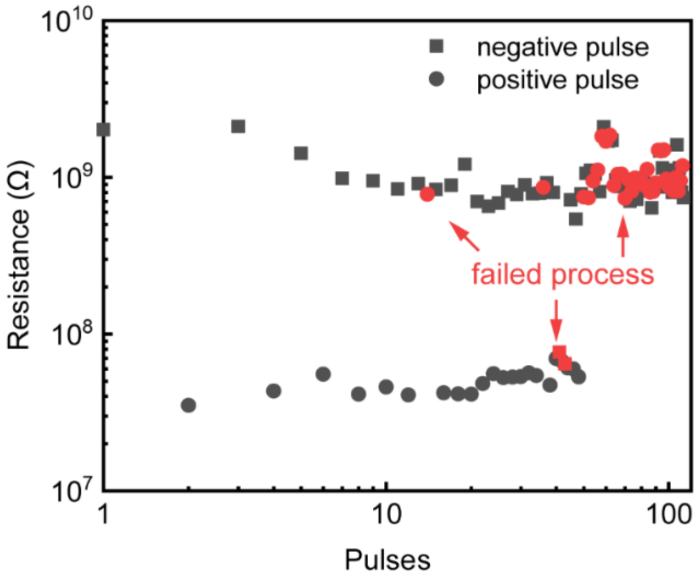

Supplementary Fig. 15 | Endurance of resistive switching with the pulses. The amplitude of the pulse is ±10 V. Read voltage ~1 V.

Materials	Dimension	DC	Times demonstrated	Pulse	Times demonstrated	Reference	Note
h-BN	2D	Y	>4	Y	11	Kenji Yasuda. et al. Science 372,1458-1462(2021)	-
h-BN	2D	Y	1	N	-	M. Vizner Stern et al.,Science372,1462-1466(2021)	-
T_d-$MoTe_2$	2D	Y	10	Y	3	Apoorv Jindal. et al. Nature 613, 48-52 (2023)	-
MoS_2/WS_2	2D	Y	about 36	Y	40-60	Lukas Rogee. et al. Science376,973-978(2022)	current leakage overtook
WSe_2	2D	Y	1 000	N	-	Yang Liu. et al. Nano Lett. 2022, 22, 3, 1265-1269	-
MoS_2	2D	Y	>5	Y	9	Astrid Weston. et al. Nat. Nanotechnol.17,390- -395 (2022)	-
WSe_2 and MoS_2	2D	Y	1	N	-	Swarup Deb. et al. Nature 612, 465-469 (2022)	-
WTe_2	2D	Y	9	N	-	Zaiyao Fei. et al. Nature 560, 336–339 (2018)	-
WSe_2, $MoSe_2$, WS_2 and MoS_2	2D	Y	>4	N	-	Xirui Wang. et al. Nat. Nanotechnol. 17, 367-371 (2022)	-
γ -InSe	2D	Y	4	N	-	Fengrui Sui. et al. Nat Commun 14, 36 (2023)	-
3R-MoS_2	2D	Y	40	N	-	Peng Meng. et al. Nat Commun 13, 7696 (2022)	-
graphene	2D	Y	20	N	-	Zhiren Zheng. et al. Nature 588, 71-76 (2020)	-
WS_2	0D	Y	10	Y	About 20	This work	-
GaSe	2D	Y	tens of times	Y	5000	Wenhui Li. et al. Nat Commun 14, 2757 (2023)	intralayer sliding

Supplementary Table 1 | Endurance of ferroelectric devices.

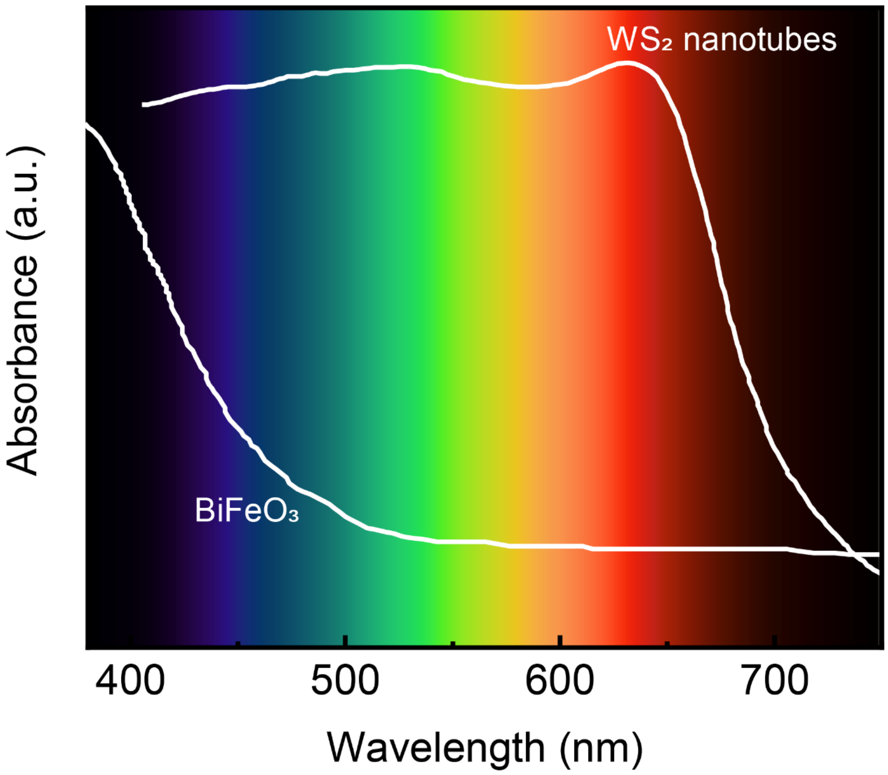

Supplementary Fig. 16 | Absorption spectra of the pristine WS_2 nanotubes and $BiFeO_3$. The result of $BiFeO_3$ is extracted from ref.[9]. The result of WS_2 is extracted from ref.[10], which is obtained with ensemble of nanotubes suspended in aqueous solution.

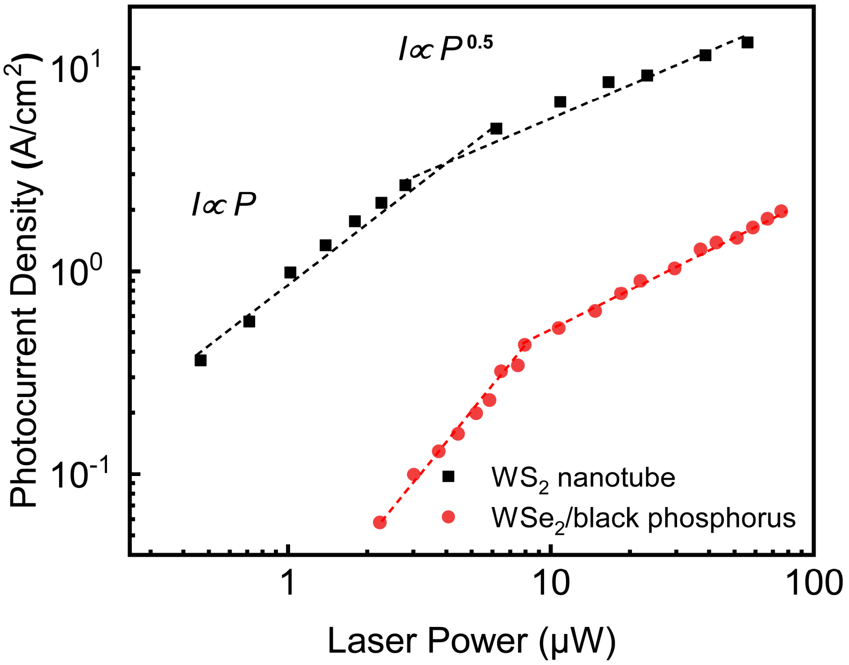

Supplementary Fig. 17 | Laser power dependence of the photocurrent density. The result of the WSe_2/black phosphorus interface was extracted from ref.[11].

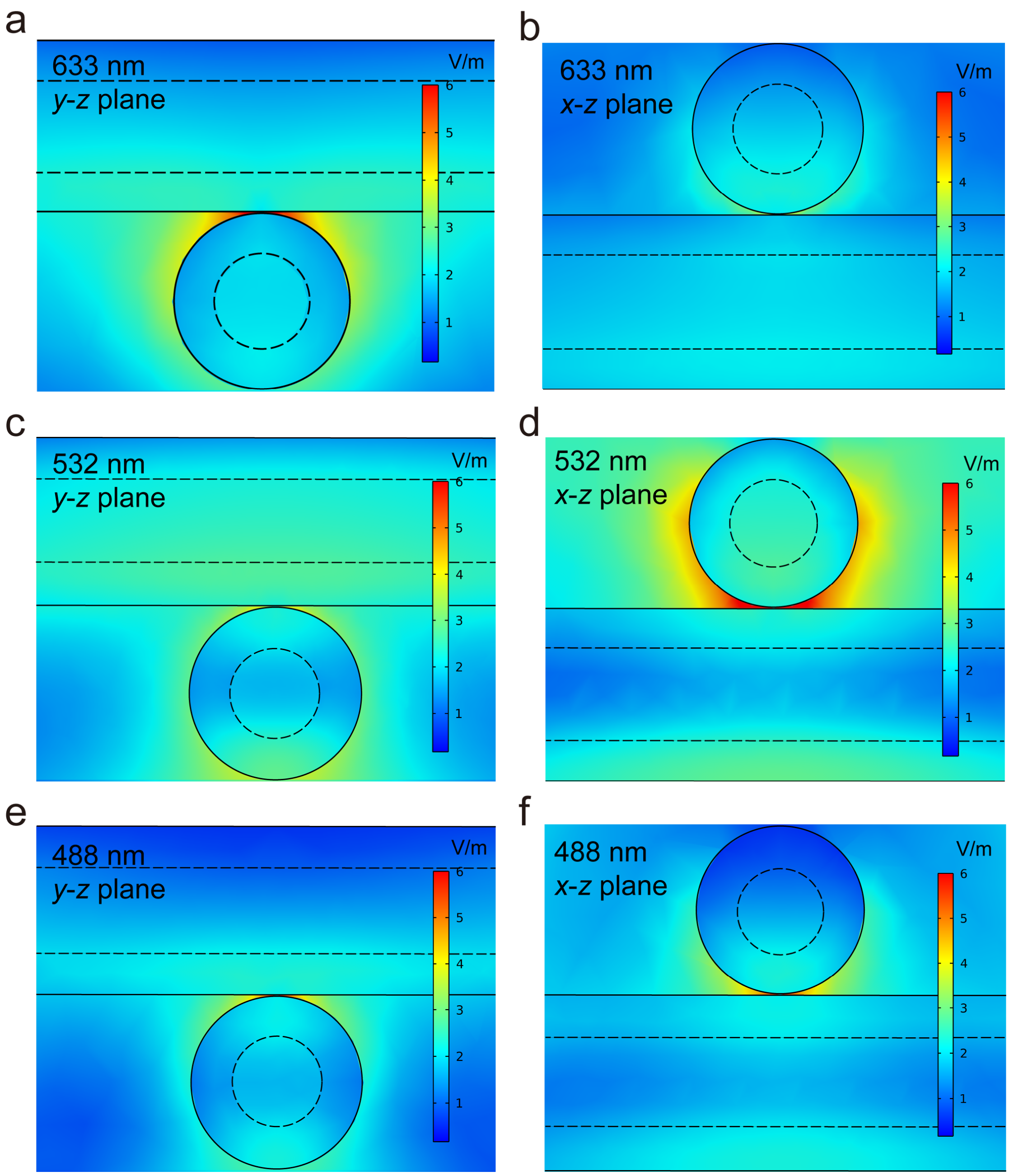

Supplementary Fig. 18 | The simulated optical electric field distribution of WS_2 nanotubes crossbar. a-f, *y-z* and *x-z* planes of WS_2 0D junction illuminated by the lasers of 633 nm(a, b), 532 nm(c, d) and 488 nm(e, f). The irradiation is from the top.

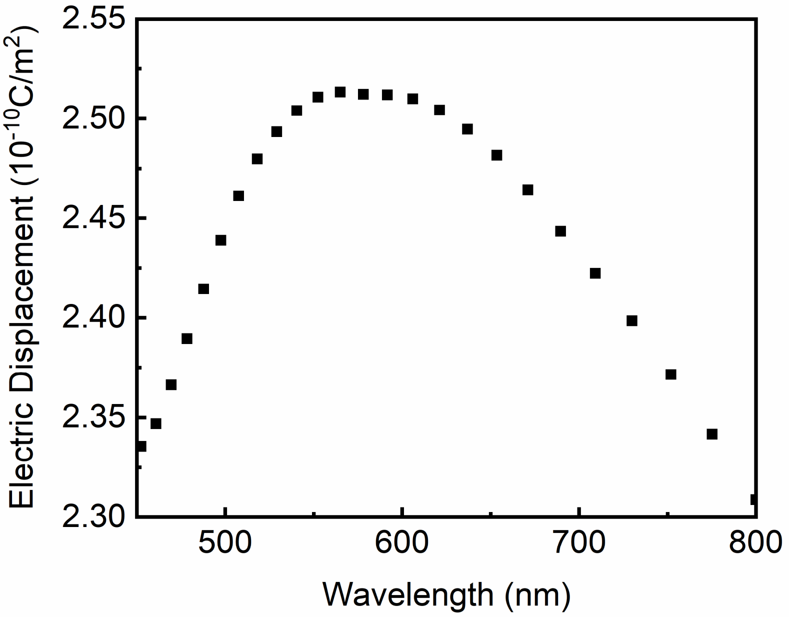

Supplementary Fig. 19 | Maximum electric displacement as a function of irradiation wavelength.

References

1 Wang, X. *et al.* Interfacial ferroelectricity in rhombohedral-stacked bilayer transition metal dichalcogenides. *Nat Nanotechnol* **17**, 367-371, doi:10.1038/s41565-021-01059-z (2022).

2 Weston, A. *et al.* Interfacial ferroelectricity in marginally twisted 2D semiconductors. *Nat Nanotechnol* **17**, 390-395, doi:10.1038/s41565-022-01072-w (2022).

3 Rogée, L. *et al.* Ferroelectricity in untwisted heterobilayers of transition metal dichalcogenides. *Science* **376**, 973-978 (2022).

4 Stern, M. V. *et al.* Interfacial Ferroelectricity by van der Waals Sliding. *Science* **372**, 1462–1466, doi:10.1126/science.abe8177 (2021).

5 Zhukov, A. *TSMC, GlobalFoundries/Samsung to present their 7nm platforms at IEDM*, 2016).

6 Park, J.-B. *et al.* Phase-change behavior of stoichiometric $Ge_2Sb_2Te_5$ in phase-change random access memory. *Journal of the electrochemical society* **154**, H139 (2007).

7 Yang, Y. *et al.* Observation of conducting filament growth in nanoscale resistive memories. *Nature communications* **3**, 1-8 (2012).

8 Choi, Y., Tsunekawa, K., Nagamine, Y. & Djayaprawira, D. Transmission electron microscopy study on the polycrystalline CoFeB⁄ MgO⁄ CoFeB based magnetic tunnel junction showing a high tunneling magnetoresistance, predicted in single crystal magnetic tunnel junction. *Journal of applied physics* **101**, 013907 (2007).

9 Arunkumar, M. *et al.* A Novel Visible Light-Driven p-Type $BiFeO_3$/n-Type SnS_2 Heterojunction Photocatalyst for Efficient Charge Separation and Enhanced Photocatalytic Activity. *Journal of Cluster Science* **32**, 1431-1439 (2021).

10 Sinha, S. S. *et al.* Size-Dependent Control of Exciton–Polariton Interactions in WS2 Nanotubes. *Small* **16**, 1904390 (2020).

11 Akamatsu, T. *et al.* A van der Waals interface that creates in-plane polarization and a spontaneous photovoltaic effect. *Science* **372**, 68-72 (2021).
