# Supplementary material for: 0D van der Waals interfacial ferroelectricity
Source: Nat Commun. 2023 Oct 31;14:5578. doi: 10.1038/s41467-023-41045-8 (PMC10618478; doi:10.1038/s41467-023-41045-8)
Supplement: Supplementary file 3 — Reporting Summary [file 41467_2023_41045_MOESM3_ESM.pdf]

Corresponding author(s): Yao Guo

Lasing Reporting Summary

Nature Research wishes to improve the reproducibility of the work that we publish. This form is intended for publication with all accepted papers reporting claims of lasing and provides structure for consistency and transparency in reporting. Some list items might not apply to an individual manuscript, but all fields must be completed for clarity.

For further information on Nature Research policies, including our data availability policy, see Authors & Referees.

Experimental design

Please check: are the following details reported in the manuscript?

1. Threshold

Plots of device output power versus pump power over a wide range of values indicating a clear threshold

- [ ] Yes
- [x] No

Not Applicable.

2. Linewidth narrowing

Plots of spectral power density for the emission at pump powers below, around, and above the lasing threshold, indicating a clear linewidth narrowing at threshold

- [ ] Yes
- [x] No

Not Applicable.

Resolution of the spectrometer used to make spectral measurements

- [ ] Yes
- [x] No

Not Applicable.

3. Coherent emission

Measurements of the coherence and/or polarization of the emission

- [ ] Yes
- [x] No

Not Applicable.

4. Beam spatial profile

Image and/or measurement of the spatial shape and profile of the emission, showing a well-defined beam above threshold

- [ ] Yes
- [x] No

Not Applicable.

5. Operating conditions

Description of the laser and pumping conditions
Continuous-wave, pulsed, temperature of operation

- [ ] Yes
- [x] No

Not Applicable.

Threshold values provided as density values (e.g. W cm^{-2} or J cm^{-2}) taking into account the area of the device

- [ ] Yes
- [x] No

Not Applicable.

6. Alternative explanations

Reasoning as to why alternative explanations have been ruled out as responsible for the emission characteristics
e.g. amplified spontaneous, directional scattering; modification of fluorescence spectrum by the cavity

- [ ] Yes
- [x] No

Not Applicable.

7. Theoretical analysis

Theoretical analysis that ensures that the experimental values measured are realistic and reasonable
e.g. laser threshold, linewidth, cavity gain-loss, efficiency

- [ ] Yes
- [x] No

Not Applicable.

8. Statistics

Number of devices fabricated and tested

- [x] Yes
- [ ] No

Line 214-215.

Statistical analysis of the device performance and lifetime (time to failure)

- [x] Yes
- [ ] No

Supplementary Fig. 14 and 15.
